# Pervasive Behavioral Effects of MicroRNA Regulation in *Drosophila*

**DOI:** 10.1534/genetics.116.195776

**Published:** 2017-05-02

**Authors:** Joao Picao-Osorio, Ines Lago-Baldaia, Pedro Patraquim, Claudio R. Alonso

**Affiliations:** Sussex Neuroscience, School of Life Sciences, University of Sussex, Brighton BN1 9QG, United Kingdom

**Keywords:** *Drosophila*, Hox genes, nervous system, CNS, behavior, microRNA, miRNA regulation

## Abstract

The effects of microRNA (miRNA) regulation on the genetic programs underlying behavior remain largely unexplored. Despite this, recent work in *Drosophila* shows that mutation of a single miRNA locus (miR-iab4/iab8) affects the capacity of the larva to correct its orientation if turned upside down (self-righting, SR), suggesting that other miRNAs might also be involved in behavioral control. Here we explore this possibility, studying early larval SR behavior in a collection of 81 *Drosophila* miRNA mutants covering almost the entire miRNA complement of the late embryo. Unexpectedly, we observe that >40% of all miRNAs tested significantly affect SR time, revealing pervasive behavioral effects of miRNA regulation in the early larva. Detailed analyses of those miRNAs affecting SR behavior (SR-miRNAs) show that individual miRNAs can affect movement in different ways, suggesting that specific molecular and cellular elements are affected by individual miRNA mutations. Furthermore, gene expression analysis shows that the *Hox* gene *Abdominal-B* (*Abd-B*) represents one of the targets deregulated by several SR-miRNAs. Our work thus reveals pervasive effects of miRNA regulation on a complex innate behavior in *Drosophila* and suggests that miRNAs may be core components of the genetic programs underlying behavioral control in other animals too.

## Abstract (precis)

Picao-Osorio *et al.* reveal pervasive effects of microRNA regulation on complex locomotor behaviors in *Drosophila* larvae: over 40% of microRNAs display...

THE cellular components underlying behavior are in one way or another affected by the activity of genes (Benzer 1967; Hotta and Benzer 1972). Through meticulous analysis of individual gene mutations and their effects on behavior it became possible to identify several genes linked to specific behaviors. These include, for instance, genes involved in circadian rhythms (*e.g.*, *period*, *timeless*, *Clock*, and *quiver*) (Konopka and Benzer 1971; Rutila *et al.* 1996, 1998; Allada *et al.* 1998; Koh *et al.* 2008), genes linked to locomotion (*e.g.*, *scribbler*, *pokey*, and *slowmo*) (Shaver *et al.* 2000; Carhan *et al.* 2003), and genes associated with geotaxis (*e.g.*, *Pdk1* and *yuri*) (Armstrong *et al.* 2006; Jones *et al.* 2009). Yet, most of this work has up to now focused on so-called protein-coding genes.

Modern genomics has unexpectedly revealed that in addition to protein-coding genes, vast sections of the genome are transcribed, producing discrete RNA transcripts that do not appear to encode functional proteins (Cech and Steitz 2014). Among this gene class of “noncoding RNAs” are the precursors for a family of short regulatory RNAs, termed microRNAs (miRNAs) able to repress protein-coding genes through the induction of messenger RNA (mRNA) degradation or the blocking of protein translation (Bartel 2009; Alonso 2012). Although estimates of the effects of miRNA repression on target gene expression are in general rather modest (Baek *et al.* 2008; Selbach *et al.* 2008; Guo *et al.* 2010), it is plausible that miRNA regulation might influence the expression of multiple gene targets simultaneously (and/or combinatorially) and that such global regulatory events could impact cellular activities underlying behavioral control. Our work explores this possibility in *Drosophila*, an excellent model system to investigate the genetic basis of behavior (Benzer 1967).

Previous work in our laboratory revealed that mutation of a single *Drosophila* miRNA (*miR-iab4*) affects a complex movement used by the larva to correct its orientation if turned upside down [self-righting (SR)], (Picao-Osorio *et al.* 2015) suggesting the possibility that other miRNAs might also be involved in behavioral control. Here we make use of the SR behavioral paradigm to establish whether other miRNAs with detectable expression in the late *Drosophila* embryo—the developmental stage at which larval neural circuitry is assembled—have impact on behavior. Unexpectedly, our data show that >40% of all miRNAs tested affect SR movement, revealing pervasive behavioral effects of miRNA regulation in *Drosophila* larva. To our best knowledge, this is the first time that miRNA regulation has been implicated at this scale in any behavior, in any animal system.

Our results added to previous studies on the effects of single miRNA mutations on other behaviors–including larval self-righting (Picao-Osorio *et al.* 2015), larval and adult feeding (Sokol and Ambros 2005; Vodala *et al.* 2012), adult climbing (Karres *et al.* 2007; Sokol *et al.* 2008; Liu *et al.* 2013; Chen *et al.* 2014; Verma *et al.* 2015), adult circadian rhythms (Luo and Sehgal 2012; Sun *et al.* 2015; Zhang *et al.* 2016), and adult startle locomotion (Yamamoto *et al.* 2008)–demonstrate that the majority of *Drosophila* miRNA mutants (55%) tested to date lead to behavioral defects of a different kind. We therefore conclude that miRNAs are key molecular regulators of the genetic programs underlying behavior in *Drosophila* and are most likely to play similar roles in other animal species too.

## Materials and Methods

### *Drosophila* strains

Flies were reared following standard procedures, at 25°, 50–60% relative humidity, and a 12-hr light/dark cycle. The *w^1118^* and *yw* stocks from the Bloomington *Drosophila* Stock Center (BDSC) were used as controls (nos. 5905 and 1495). The miRNA knockout stock collection was also obtained from the BDSC (Supplemental Material, File S1 and File S5). The miRNA mutant collection was generated and deposited in BDSC by Stephen Cohen’s laboratory (Chen *et al.* 2014). The following stocks were also used: *Abd-B^LDN^-Gal4* and *Abd-B^199^-Gal4* (de Navas *et al.* 2006), and *UAS-Abd-B(m)* (Castelli-Gair *et al.* 1994), all kindly given by Ernesto Sánchez-Herrero (Centro de Biología Molecular Severo Ochoa, Universidad Autónoma de Madrid, Spain); *elav^c155^-Gal4* (no. 458), *Mef2-Gal4* (no. 27390), *UAS-Scramble-SP* (no. 61501), *UAS-miR-980-SP* (no. 61465), *UAS-miR-8-SP* (no. 61374), and *UAS-miR-278-SP* (no. 61409) (Fulga *et al.* 2015), and 10xUAS-IVS-GFP-WPRE (no. 32202) were obtained from the BDSC; and *tubulin-Gal4* (Lee and Luo 1999).

### Behavioral analyses

All flies were kept in small collection cages with apple juice agar plates supplemented with yeast paste at 25°, except flies for miRNA-sponge experiments that were maintained at 29° to increase GAL4 activity. Embryos were collected from these plates and aged until stage 17 (Campos-Ortega and Hartenstein 1985) in humid chambers at 25°. Note that embryos for miRNA-sponge experiments were reared at 29° to increase GAL4 activity. Freshly hatched first instar larvae (<30-min posthatching) were placed on 1.5% agar plates and allowed to acclimatize for 1 min. All behavioral assays were performed as “blind tests” in respect to their genotype (including controls): *miRNA* mutants showing SR defects were assayed in at least three independent experiments by two experimentalists in parallel. The SR test was performed as previously described (Picao-Osorio *et al.* 2015). Briefly, freshly hatched larvae were gently rolled over with a rounded micro needle to an inverted position (ventral denticle belts up) and the time to return to the noninverted position (dorsal longitudinal trachea up, original position) was measured, to a maximum of 5 min. To ensure consistency across experiments humidity levels on agar plates were carefully adjusted prior to the experiment until control “wild type” larvae displayed SR times in the order of 8–10s. Ten to 70 larvae were analyzed per genotype. We estimated that the minimum sample size for a given miRNA mutant not having a significant delay of self-righting (type II error, false negative) is nine larvae per genotype to detect a twofold difference in SR (16.342 sec to SR; *i.e.*, an 8.171-sec difference) for an α = 0.05 and power of 0.9 (β = 0.1); given that *σ^WT^* = 3.687, $\bar{x}$*^WT^* = 8.171 and effect size = 2.216 (Faul *et al.* 2007, 2009). We used a minimum of 10 larvae and a maximum of 69 larvae (average of 17 larvae) per genotype in the miRNA mutants that did not show a significant self-righting delay in at least two independent experiments. We estimated that the minimum sample size for a given miRNA mutant to show a significant delay of self-righting (type I error, false positive) is 22 larvae per genotype to detect a twofold difference in SR (16.342 sec to SR) for an α = 0.0006 (*P*-value after Bonferroni correction; 0.05/8 = 0.0006) and power of 0.9 (β = 0.1); given that *σ^WT^* = 3.687, $\bar{x}$*^WT^* = 8.171 and effect size = 2.216 (Faul *et al.* 2007, 2009). We used a minimum of 22 larvae and a maximum of 66 larvae (average of 46 larvae) per genotype in the miRNA mutants that showed a significant self-righting delay in at least three independent experiments by two experimentalists in parallel. Behavioral videos are available upon request. The SR sequence of movements was analyzed with the open source software 1.2 VCode (http://social.cs.uiuc.edu/projects/vcode.html). To quantify the average duration of movements during SR, the time spent on each of the four movements described in Figure 2A was extracted from VCode. For the touch response (TR) test, embryos were transferred directly to 1.5% agar plates and tested within the first hour posthatching by only one experimentalist to maintain touch consistency. A soft stroke at the anterior region was performed with an eyelash, and the sequence of movements of the response was scored: no response = 0, hesitation = 1, withdraws anterior = 2, single backward wave and/or turn = 3, multiple backward waves = 4 [Figure 2D, according to Kernan *et al.* (1994)]. Fifteen to 33 larvae were analyzed per genotype. We estimated that the minimum sample size for a given miRNA mutant to show a significant difference of 1 in TR score is 11 larvae per genotype, for an α = 0.05 and power of 0.9 (β = 0.1); given that *σ^WT^* = 0.4469, $\bar{x}$*^WT^* = 3.194 and effect size = 2.238 (Faul *et al.* 2007, 2009). We used a minimum of 15 larvae and a maximum of 33 larvae (average of 22 larvae) per genotype, in at least two independent experiments. All behavioral experiments were conducted at 25° and recorded with a Leica DFC 340 FX camera mounted on a Leica M165 FC microscope. Crawling speed of freely moving larvae was recorded using the FIM-table setup (Risse *et al.* 2013) and analyzed with the FIMtrack software (Risse *et al.* 2014). Accumulative distance traveled was extracted in 11–22 larvae per genotype in at least two independent 2-min videos. The statistical analyses of the SR and TR screens were performed using the nonparametrical Mann–Whitney *U*-test with Bonferroni correction for multiple comparisons. The correlation between SR time and peristaltic waves per minute was analyzed using the Spearman correlation. Statistical analyses were executed in the Prism GraphPad 6.0 software package.

### Bioinformatic analyses

#### miRNA seed sequence analyses:

All sequences for the mature miRNA complement of *Drosophila melanogaster* were first retrieved from miRBase.org (release 21, June 2014). These sequences were then trimmed to include only nucleotide positions 2–7 (the miRNA “seed”). A dissimilarity matrix was then obtained for all *Drosophila* miRNA seed sequences using the daisy R package (Gower distance). This matrix was used to hierarchically cluster all *D. melanogaster* miRNAs based on seed similarity, using the hclust R package (Ward’s *D* method). This produced a dendrogram representing the known *D. melanogaster* seed space. The SR miRNA seeds were then graphically highlighted within the context of the *Drosophila* miRNA seed space using the R packages dendextend and circlize. To compare the observed distribution of SR miRNA seeds with a null hypothesis, the aforementioned dendrogram was divided in three portions of equal seed-space coverage, using the *k*-means cluster determination method. The seed distribution of SR-miRNAs was obtained by counting the number of mature SR-miRNAs that fell within each of the three clusters, translated into percentages. This was compared to the distribution (in percentage) of all mature *Drosophila* miRNAs, using Pearson’s χ^2^-test.

#### miRNA target predictions:

The longest BDGP6-annotated 3′-UTR sequences of the three posterior *Hox* genes, *Ubx*, *abd-A*, and *Abd-B*, were used for miRNA target predictions. Predictions were performed locally using both PITA (Kertesz *et al.* 2007) and miRanda (Betel *et al.* 2008) software packages, applying default settings in both tools. To increase confidence in the predicted targets, PITA predictions were filtered using the advised cut-off threshold of ΔΔG ≤ −10, and overlapped with miRanda predictions.

### Immunocytochemistry and fluorescent *in situ* hybridization

Late stage-16 embryos were collected, fixed, and immunostained following standard protocols. Primary antibodies used were monoclonal mouse anti-Ubx (FP3.38, Developmental Studies Hybridoma Bank; 1:20), mouse anti-Abd-B (1A2E9, Developmental Studies Hybridoma Bank; 1:20), goat anti-Abd-A (dH-17, Santa Cruz Biotechnology, Dallas TX; 1:20) and rabbit anti-GFP (A6455, Molecular Probes, Eugene, OR; 1:750). Secondary antibodies used were anti-mouse-A488, anti-mouse-Alexa 555, anti-goat-A555, anti-rabbit-A555, and anti-rabbit-A488 (1:750, Molecular Probes). All embryos were counterstained with 4′,6-diamidino-2-phenylindole (DAPI) to label nuclei and mounted in Vectashield antifade medium (Vector Laboratories, Burlingame, CA). A Leica SP8 confocal microscope was used for fluorescent imaging, and images were processed and analyzed using ImageJ and Adobe Photoshop. Expression analysis of fluorescent immunostainings along the anterior–posterior (A–P) axis was done on ImageJ. Briefly, confocal stacks of the ventral nerve cord of each specimen were collapsed into one projection (sum slices), and fluorescent intensity (Gray value) of Hox expression was measured with the Plot profile tool. The fluorescent intensity of each specimen was normalized by the background expression in the CNS (*i.e.*, a region of the CNS where the respective *Hox* gene is not expressed). The entire immunocytochemistry protocol (embryo collection, fixation, immunostaining, specimen mounting, and imaging) was done in parallel for controls and mutants, and confocal images were collected, applying the same settings. Protein expression was quantified in at least 10 embryos per genotype for each immunostaining (as in Rogulja-Ortmann *et al.* 2014; Crocker *et al.* 2016). Immunostainings were carried out in triplicates.

Fluorescent RNA *in situ* hybridizations (FISHs) for the primary RNA transcripts of *miR-980* (ewg), *miR-8* (*ncRNA:CR43650-RA*), and *miR-278* (*CG42524-RC*) were performed as described previously in Rogulja-Ortmann *et al.* (2014). Templates of RNA probes were obtained from PCR-amplified embryonic complementary DNA (cDNA) with the following primers: 5′-CAGCCAAAGGAGTTCGACTG-3′ and 5′-CATCCATCCTCACATTGGCC-3′ for *miR-980* (ewg); 5′-ATATGTGTGCGGGCGTTATT-3′ and 5′-GATCTAATGCTGCCCGGTAA-3′ for *miR-8* (*ncRNA:CR43650-RA*); and 5′-CGAAAACGATGGTGAGAGGG-3′ and 5′-TCGTTGACAAATGGCGTTACA-3′ for *miR-278* (*CG42524-RC*) and cloned into pGEM-T easy vector (Promega, Madison, WI). RNA probes were labeled with digoxigenin (DIG) using the RNA Labeling Kit (Roche, Indianapolis, IN) according to the manufacturer’s instructions. Fluorescent detection of RNA probes was done using anti-DIG-POD (1:500, Roche) followed by the Cy3 TSA amplification kit (1:50, Perkin Elmer). Embryos were mounted and imaged as described before.

### Data availability

All fly strains and reagents are available upon request. All data presented in this study are included in the article or in Supplemental Material.

## Results and Discussion

**Figure 1:**
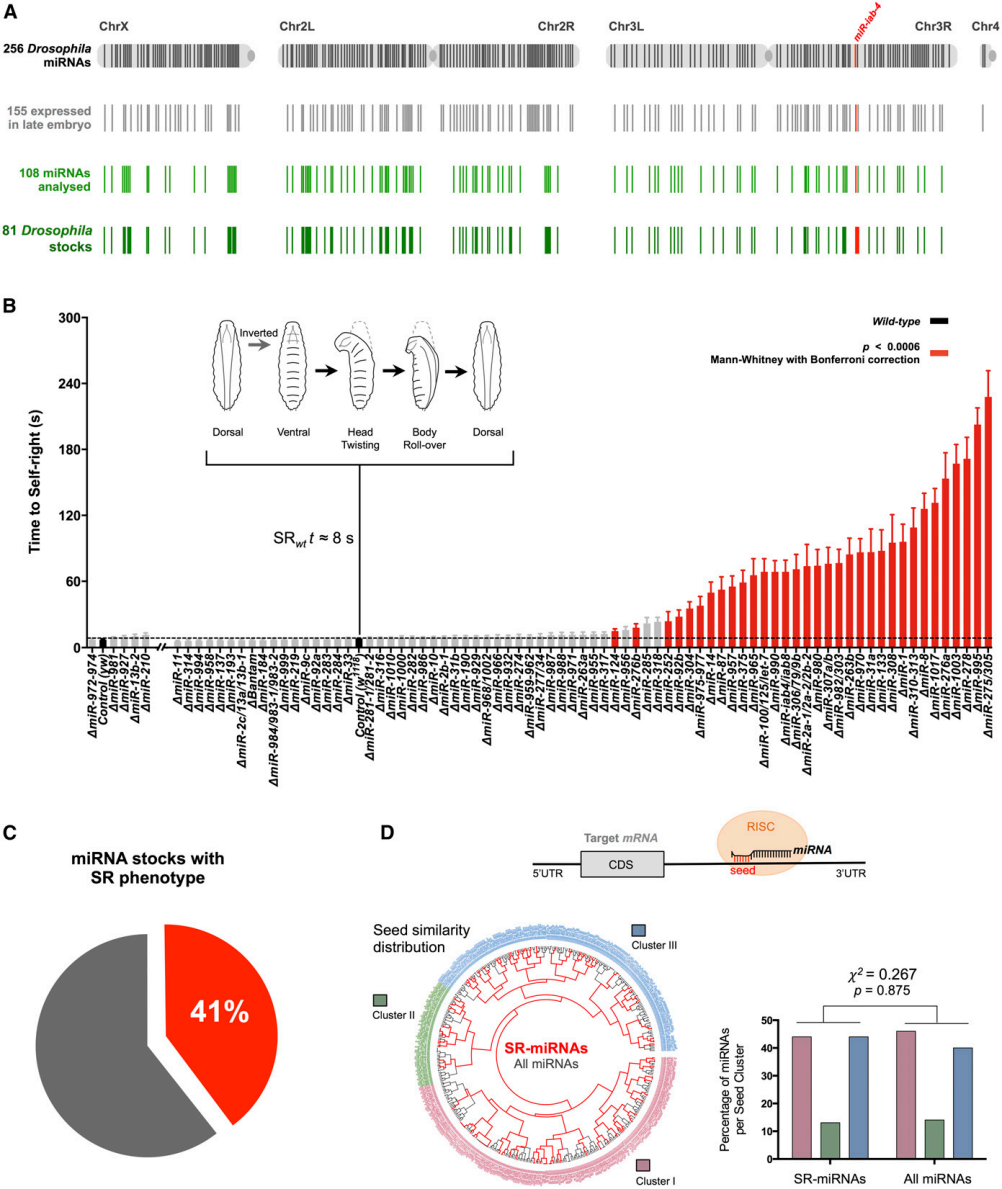
Pervasive role of miRNAs in self-righting behavior. (A) Graphic representation of the miRNA precursor sequences along the four *Drosophila* chromosomes. The total 256 miRNA precursor sequences from the latest miRBase version (miRBase 21) (Kozomara and Griffiths-Jones 2014) are represented in black lines. The 155 miRNAs expressed in late embryos are represented in gray lines (data accessible at National Center for Biotechnology Information GEO database (Barrett *et al.* 2013) accession no. GSM364902; 12- to 24-hr *Drosophila* embryos). Represented in green are the 108 individual miRNA precursors (light green lines) included in the 81 mutant stocks analyzed (dark green lines) (File S1 and File S5). In red is the *miR-iab-4* that had been previously described to disrupt SR (Picao-Osorio *et al.* 2015). (B) Quantification of the time required for successful completion of self-righting (mean ± SEM; with an average of 29 larvae per genotype). The two different *miRNA* mutant genetic backgrounds—*yw* and *w^1118^*—were compared with the respective controls (in black) and are separated in two groups: *yw* and five mutants and *w^1118^* and 76 mutants. The mutants showing SR delay with statistical significance of *P* ≤ 0.0006 (Mann–Whitney *U*-test with Bonferroni correction) are depicted in red. Representation of the sequential movements of SR is depicted above: when placed in an inverted position (ventral up), larvae twist their heads and roll their bodies onto their ventral surface (dorsal up). This sequence takes an average of 8 sec in control (wt) larvae. (C) Pie chart of the percentage of miRNA mutant stocks (33 out of 81) showing a statistically significant delay in SR time. (D, top) Diagram illustrating the miRNA-mediated downregulation of gene expression through association with the RNA-induced silencing complex (RISC) and seed pairing with a 3′-UTR target. (D) Hierarchical clustering of the *D. melanogaster* miRNA seed sequence complement (positions 2–7, gray). The distribution of SR-miRNA seed sequences (red) within the *Drosophila* miRNA seed-sequence space (black) is identical to the distribution of all *Drosophila* mature miRNAs (right, Pearson’s χ^2^-test = 0.267, *P* = 0.875).

We applied a high-throughput behavioral genetic approach that establishes SR times for a collection of 81 null miRNA mutants (Figure 1, A and B and File S1 and File S5) (Chen *et al.* 2014), which represent ∼90% (*i.e.*, 89.5%) of all the miRNAs detected by RNA sequencing in the late embryo (File S2 and File S5) (Chung *et al.* 2008). Remarkably we observe that >40% of all miRNA mutants tested (*i.e.*, 33/81; 40.74%) significantly delayed SR response (Figure 1, B and C, Mann–Whitney *U*-test with Bonferroni correction, *P* < 0.0006). These data show that genetic ablation of a large number of miRNAs expressed in the late *Drosophila* embryo affect SR, implying a pervasive role of miRNA regulation on a complex innate behavior in *Drosophila*.

Of all miRNA mutants affecting SR (termed *SR-miRNA*s) some displayed striking effects on SR time (*e.g.*, Δ*miR-8*, Δ*miR-1017*, Δ*miR-276a*, and Δ*miR-1003*), while others showed modest, yet statistically significant effects (*e.g.*, Δ*miR-276b*, Δ*miR-252*, Δ*miR-92b*, and Δ*miR-304*) (Figure 1B). A potentially trivial reason underlying miRNAs effects on SR timing is that highly expressed miRNAs have higher impact on SR than other miRNA species with lower expression levels. If this were true we should expect to observe a positive correlation between miRNA expression level and SR time. In contrast with this prediction, we observed no significant correlation between miRNA expression and SR time (Spearman correlation *r* = 0.0735; *P* = 0.5146; Figure S1 in File S4, File S2, and File S5), suggesting that expression level *per se* does not explain the nature of observed miRNA effects on SR behavior.

The diversity of effects detected in our SR tests suggested that individual miRNAs might affect the development and/or function of the neural circuits underlying SR through diverse molecular and cellular effects. If this were true, SR-miRNAs are expected to display distinct rather than common features in regards to recognition of target mRNAs. As a first step in the investigation of this problem, we considered the possibility that SR-miRNAs might possess identical recognition sequences (seeds) involved in target interaction. To explore this possibility, we developed a computational clustering approach to determine whether SR-miRNAs shared common core sequence elements involved in target gene recognition (Figure 1D). This approach revealed that SR-miRNA seeds map to diverse branches scattered around an unrooted phylogenetic tree, producing a pattern that is indistinguishable from a random distribution (Figure 1D, Pearson’s χ^2^-test = 0.267, *P* = 0.875), demonstrating that SR-miRNAs do not act via identical miRNA–mRNA interaction motifs. This analysis, however, does not exclude the possibility that some SR-miRNAs may interact with common mRNA targets via miRNA-specific target sites (see below).

**Figure 2:**
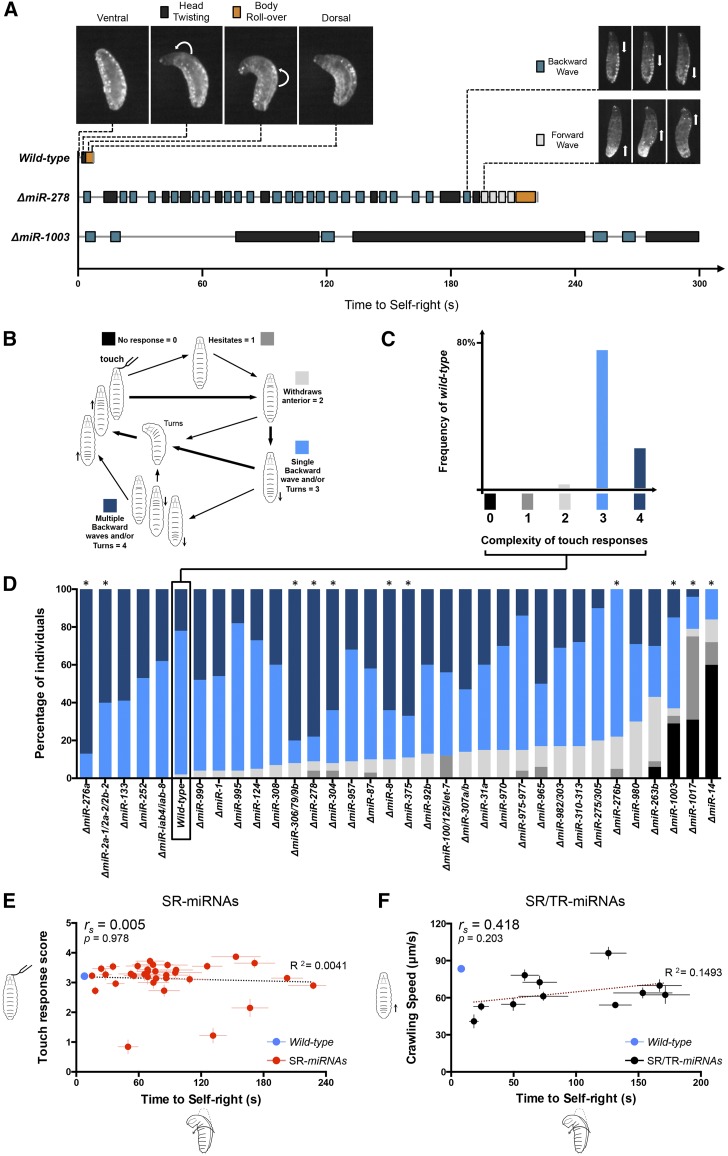
Diversity of miRNA effects on SR. (A, top left) Representative wild-type SR sequence in a single larva with two main phases: head twisting (black) and body roll-over (orange). (A, top right) Representation of other SR-miRNA movements while in an inverted position during SR struggles: backward (blue) and forward (light gray) waves. (A, bottom) Representative examples of SR sequences in single miRNA mutant larvae, depicting the occurrence of different movements and their frequency and duration. *miR-278* mutant larvae are active, frequently alternating between different movements (*e.g.*, forward and backward waves) until they are able to roll over their bodies. Δ*miR-1003* larvae generally take a longer time performing each SR phase. More SR-miRNA mutant examples are described in Figure 4D. (B) Representation of the different responses to touch in the anterior region and respective scores (0–4) based on Kernan *et al.* (1994) (TR). Insensitivity to touch is shown in black (score 0). Simpler and more complex responses are represented in shades of gray and blue, respectively, and are translated in scores ranging from 1 to 4. (C) Frequency of the different responses to touch in wild-type larvae. Simpler responses (shades of gray) are extremely rare. While around 20% of the individuals show more complex responses (with multiple backward waves), the most common response (∼80% of the cases) is obstacle avoidance through turning or performance of a single backward wave followed by turning. (D) Percentage of the different TR scores in each miRNA mutant (*N* = 15–33 larvae per genotype). * represents significant deviations from the wild-type responses (*w^1118^*, black box) with *P* < 0.0015 (Mann–Whitney *U*-test with Bonferroni correction). (E) Correlation between TR scores (*y*-axis) and time to SR (*x*-axis) of all 33 SR-miRNAs. The wild-type genotype is depicted by a blue circle, the SR-miRNAs by red circles, and linear regression in dotted black line (*R*^2^ = 0.0041). The Spearman coefficient (*r*_s_) and *P*-value are shown. There is no significant correlation between TR and the SR delay (*r*_s_ = 0.005; *P* = 0.978). (F) Correlation between crawling speed (micrometers per second) of freely exploratory larvae behavior (*y*-axis) (mean ± SEM; with an average of 17 larvae per genotype) and time to self-right (*x*-axis) of the 11 mutants showing both SR and TR phenotypes (SR/TR-miRNAs, black circles). Wild type is depicted by a blue circle. Linear regression line in dark red (*R*^2^ = 0.1493). The Spearman coefficient (*r*_s_) and *P*-value are shown. There is no significant correlation between crawling speed and the SR delay (*r*_s_ = 0.4182; *P* = 0.203).

Although SR time proved to be a valuable unidimensional variable to score for SR defects in miRNA mutants, on its own, it is unlikely to capture the full spectrum of behavioral contributions of each mutant to the SR sequence. To gain insight into the specificity by which each miRNA affected SR, we analyzed SR movement using video recordings seeking to establish the ways in which the SR sequence observed in wild-type specimens was modified by individual miRNA mutations (Figure 2A and Figure 4). Wild-type larvae have a stereotypical SR sequence (Figure 2A, top) (Ball *et al.* 1985; Picao-Osorio *et al.* 2015): when inverted (“ventral up”) larvae twist their heads (“head twisting”) and almost immediately roll over their bodies (“body roll-over”) to restore a normal position (“dorsal up”). In contrast, SR-miRNA mutants display a great variety of behavioral responses in SR behavior (Figure 2A, bottom). For instance, some mutants appear as languid or “sluggish” and trigger slow bouts of backward peristaltic waves and head twisting movements (*e.g.*, *miR-1003*, *miR-1017*, and *miR-87*), while other mutants develop trains of perilstaltic waves combined with head twisting moves (*e.g.*, *miR-278*, *miR-8*, and *miR-980*) (Figure 2A; see also Figure 4).

To further explore the specificity of the behavioral effects observed in SR miRNAs, we looked at another previously described larval behavior: touch response (TR) (Kernan *et al.* 1994). The value of TR analysis is that it concerns—at least to a substantial degree—a different anatomical aspect of the larva than SR (*i.e.*, anterior mechanosensation) (Kernan *et al.* 1994; Zhou *et al.* 2012), allowing us to look for effects of miRNA mutation on a different and complex innate larval movement. To establish the capacity of miRNA mutants to deliver a full or impaired TR sequence, we used a previously described scoring system (Kernan *et al.* 1994) applying a scale that ranges from 0 to 4 (0 for no response, 4 for a complex response) (Figure 2, B–E). When this system is applied to wild-type stocks, a distribution of responses is obtained, with most individuals exhibiting scores of 3 (Figure 2, C and D). Analysis of TR sequences in the 33 mutant stocks that displayed SR defects revealed that 33% of them showed TR problems (Mann–Whitney *U*-test with Bonferroni correction, *P* < 0.0015) while the remaining 66% did not show any effects on TR (Figure 2D). The fact that the majority of the miRNAs affecting SR (66%) did not affect TR shows that most SR mutants are not impaired in their general capacity to engage in complex behaviors. Furthermore, we detected the absence of any correlation between TR and SR time (Spearman correlation *r*_s_ = −0.005; *P* = 0.978; Figure 2E), and there is no significant correlation between crawling speed and SR behavior (*r*_s_ = 0.4182; *P* = 0.203; Figure 2F), or when comparing crawling speed to TR (*r*_s_ = 0.4455; *P* = 0.173; data not shown) for the 11 miRNA mutants showing both SR and TR phenotype. These results provide further support to the notion that SR-miRNAs exert their effects on SR through diverse mechanisms and suggest that miRNA effects on SR are not the result of broad “nonspecific” pleiotropic roles of miRNAs on behavioral processes.

The behavioral effects of miRNA mutation that we observe here may be explained by two plausible scenarios. One, is that normal miRNA expression is required for the correct formation of the neural and neuromuscular networks underlying SR conforming to what might be called a “developmental” role of the miRNA. Another, is that miRNA activity is linked to normal physiological functions in affected cells. At this point in time, we see no reason to consider that these scenarios should be mutually exclusive.

To advance the understanding of the molecular basis underlying the effects of this large number of miRNAs, we investigated the effects of SR-miRNAs on the expression of the posterior *Hox* genes, including all three members of the *Bithorax Complex* (*BX-C*): *Ultrabithorax* (*Ubx*), *abdominal-A* (*abd-A*), and *Abdominal-B* (*Abd-B*) (Sanchez-Herrero *et al.* 1985; Tiong *et al.* 1985; Mallo and Alonso 2013). These genes were of particular interest to us due to several reasons. First, the *Hox* genes encode a family of transcription factors expressed in different tissues—including the CNS—at particular coordinates along the body axis (Maeda and Karch 2009; Mallo and Alonso 2013). Due to their restricted axial expression, the *Hox* genes mold the process of neuronal differentiation in a segment-specific fashion, aligning the developing CNS to regional muscle networks (Rogulja-Ortmann and Technau 2008; Rogulja-Ortmann *et al.* 2014). Given that the SR sequence involves the coordinated contraction of consecutive body segments of the larva, changes in the expression of these developmental regulators represented a particularly attractive biological scenario that could link miRNA regulation to SR. Second, our previous analysis of the behavioral impact of *Drosophila miR-iab4* led us to the discovery that this individual miRNA triggered its effects on SR via repression of *Ubx* (Picao-Osorio *et al.* 2015), making it plausible that other miRNAs affecting SR may also act via this same regulator. Third, the abdominal region of the early first instar larva, known to be patterned by posterior *Hox* genes (Lewis 1978; Maeda and Karch 2006), underlies almost the entire anatomy of the individual at this stage and is directly involved in the SR sequence (Picao-Osorio *et al.* 2015). Fourth, *BX-C* genes control the formation of the neuromuscular network that coordinates locomotion in *Drosophila* larvae (Dixit *et al.* 2008). Based on these considerations, we decided to experimentally test whether posterior *Hox* genes were derepressed in SR-miRNAs.

To narrow down the scope of the expression study from several dozens of miRNAs to a smaller more manageable subset of mutants, we considered the likelihood that miRNAs may interact with *BX-C* gene transcripts (see File S3 and File S5 and *Materials and Methods*) as well as previous information regarding neuronal roles for candidate miRNAs. Based on this, we selected six miRNAs for detailed study: *miR-278* (Teleman *et al.* 2006), *miR-1003* (Brown *et al.* 2014), *miR-8* (Aboobaker *et al.* 2005; Karres *et al.* 2007), *miR-310c* (Tsurudome *et al.* 2010), *miR-980* (Marrone *et al.* 2012; Guven-Ozkan *et al.* 2016), and *miR-iab4/iab8* (Bender 2008; Tyler *et al.* 2008; Thomsen *et al.* 2010; Picao-Osorio *et al.* 2015).

**Figure 3:**
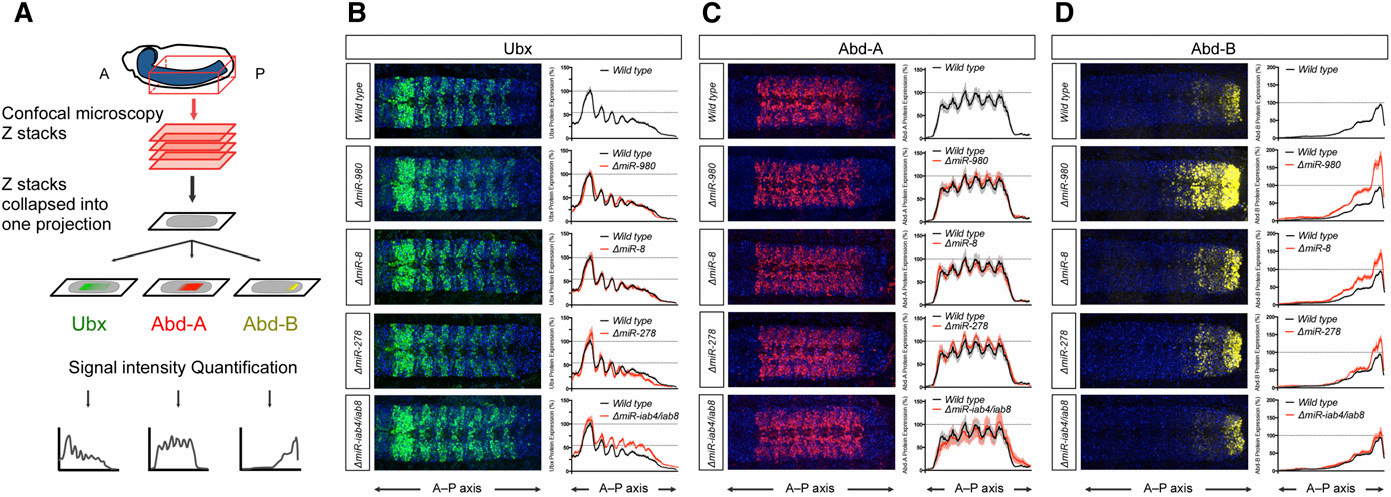
SR-miRNAs control *Hox* gene expression. (A) Schematic representation of Hox protein expression analysis along the A–P axis (see *Materials and Methods*). Immunostained whole-mounted embryos for the three BX-C proteins were imaged using confocal microscopy. Confocal stacks of the ventral nerve cord of each specimen were collapsed into one projection and levels of expression along the A–P axis quantified. (B–D, left) Embryonic protein expression of Ubx (B, green), Abd-A (C, red), and Abd-B (D, yellow) in the ventral nerve cord of wild-type and mutants for *miR-980*, *miR-8*, *miR-278*, and *miR-iab-4/iab-8* at late 16 stage. (B–D, right) Profile quantification along the A–P axis for the three Hox proteins in the wild type (mean in black line and SEM in gray) and in the *miRNA* mutants (mean in red line and SEM in lighter red). (B) Only Δ*miR-iab-4/iab-8* shows differences in Ubx protein expression (as previously described by Bender 2008; Thomsen *et al.* 2010; Picao-Osorio *et al.* 2015). (C) No significant expression change was observed in Abd-A protein in the four *miRNA* mutants. (D) Significant expression change in Abd-B protein for *miR-980*, *miR-8*, and *miR-278* mutants. *N* = 10 embryos per genotype for each immunostaining. DAPI is in blue. Anterior is to the left.

To determine whether the expression patterns of all three posterior Hox proteins: Ubx, abd-A, and Abd-B, was increased (derepressed) in the late embryonic nervous system of the selected SR-miRNAs, we applied a combination of immunocytochemistry and confocal imaging followed by a quantitative method that allows accurate comparison of gene expression patterns within the late embryonic CNS across individuals previously developed in our laboratory (Rogulja-Ortmann *et al.* 2014) (Figure 3 and Figure S2 in File S4). These experiments revealed that mutations disrupting the expression of three miRNAs: *miR-8*, *miR-980*, and *miR-278*, led to significant changes in the expression of one of the Hox proteins examined: Abd-B (Figure 3D). Although bioinformatic predictions do indeed suggest that *Abd-B* contains high-affinity target sites for these three miRNAs (Figure S4E in File S4), we wish to note that our experiments do not allow us to determine whether the effects on Abd-B expression emerge from direct or indirect interactions between these SR-miRNAs and Abd-B sequences. Effects on Ubx expression were only detected in *miR-iab4/iab8* mutants in line with previous findings (Figure 3B and Figure S2A in File S4) (Bender 2008; Thomsen *et al.* 2010; Picao-Osorio *et al.* 2015); no expression changes were detected in neural Abd-A protein patterns (Figure 3C and Figure S2B in File S4).

**Figure 4:**
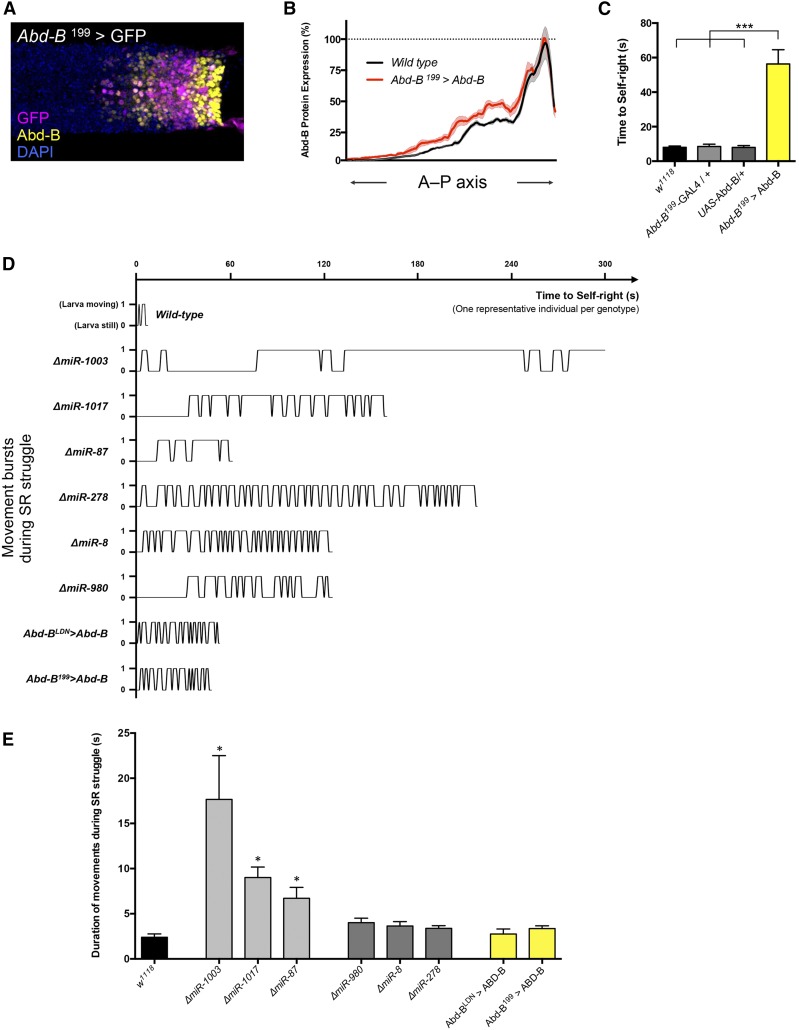
Overexpression of Abd-B disrupts SR behavior. (A) Expression pattern of *Abd-B^199^*-GAL4 driver (GFP, magenta) with respect to the endogenous pattern of Abd-B protein expression (yellow) in dissected embryonic ventral nerve cord. DAPI is in blue and anterior is to the left. (B) Quantification of Abd-B expression profile along the A–P axis in dissected embryonic nerve cords of wild-type (*w^1118^*, mean in black and SEM in gray) and Abd-B overexpression (*Abd*-B^199^ > Abd-B, mean in magenta and SEM in light magenta) (*N* = 9 embryos per genotype). (C) Significant delay in time to SR in larvae overexpressing Abd-B (*Abd-B^199^* > Abd-B, yellow bars) in comparison with wild-type (*w^1118^*) and parental lines (*Abd-B^199^*-GAL4/+, light gray bar, and *UAS*-Abd-B/+ in dark gray) (mean ± SEM; an average of 20 larvae per genotype were analyzed; Mann–Whitney *U*-test with Bonferroni correction, *** *P* < 0.001). (D) Representative examples of activity patterns of SR struggle of single miRNA mutant larvae and Abd-B overexpressing larvae. During the SR routine, we assigned a value of 1 if the larva performed any of the movements mentioned in Figure 2A, and 0 when it remained still. Mutants *miR-1003*, *miR-1017*, and *miR-87* represent examples of SR-miRNA mutants that take a longer to perform each SR phase. In contrast, mutant larvae for *miR-278*, *miR-8*, and *miR-980* are examples of active larvae that frequently alternate between different movements (*e.g.*, forward and backward waves) until they are able to roll over their bodies. The characteristic SR struggle of Abd-B overexpressing larvae (*Abd*-B^LDN^ > Abd-B and *Abd*-B^199^ > Abd-B) was comparable to *miR-278*, *miR-8*, and *miR-980*. (E) Quantification of duration of each movement during SR behavior. SR-miRNA mutants *miR-1003*, *miR-1017*, and *miR-87* (light gray bars) showed longer periods on each SR movement compared to the wild type (*w^1118^*, black bar). Conversely, SR-miRNA mutants *miR-278*, *miR-8*, and *miR-980* (dark gray bars) and larvae overexpressing Abd-B (yellow bars) showed similar duration on each SR movement compared to the wild type (mean ± SEM; *N* = 4 larvae; Mann–Whitney *U*-test, * *P* < 0.05).

The discovery that several SR-miRNAs derepressed Abd-B expression suggested the possibility that overexpression of this Hox protein might be sufficient to trigger a SR phenotype, phenocopying the effects of miRNA mutation. To explore this hypothesis, we used a previously described Gal4-driver line that partially mimics the expression pattern of Abd-B within the nervous system: *Abd-B^199^*-Gal4 (de Navas *et al.* 2006). Detailed comparison of the activity of these *Abd-B*-Gal4 drivers (by means of UAS-GFP expression) with the endogenous pattern of expression of the Abd-B protein (Figure 4, A and B and Figure S5C in File S4) revealed that this driver line only recapitulates Abd-B expression across a limited region of the CNS in the posterior abdomen (parasegments 12 and 13). Despite these limitations, Gal4-mediated increase in Abd-B protein levels led to a very clear SR phenotype (Mann–Whitney *U*-test with Bonferroni correction, *P* < 0.001), suggesting that changes in the expression of Abd-B might contribute to the mechanisms that link SR-miRNAs to the SR phenotype (Figure 4C). Similar results were obtained with another independent *Abd-B-Gal4* driver (*Abd-B^LDN^*-Gal4) (de Navas *et al.* 2006), providing further support to the likely roles of *Abd-B* in SR (Figures S3 and S5B in File S4). Notably, the behavioral phenotypes induced by Abd-B overexpression result in phenotypes of similar kind to those observed in miRNA mutants *miR-980*, *miR-8*, and *miR-278*: in all these conditions—but not in the case of other miRNA mutants—larvae develop a series of rapid “bursts” of activity while seeking to return to their normal orientation in SR tests (Figure 4, D and E). These experiments, added to previous work in our laboratory (Picao-Osorio *et al.* 2015), suggest that miRNA-dependent modulation of *Hox* gene activity might play a central role in the acquisition of neural functions with impact on behavior and open up an opportunity to investigate the molecular roles of miRNAs and *Hox* genes during the specification of neuronal physiology and development in *Drosophila*. We are currently investigating this problem at the mechanistic and cellular levels, focusing on the roles played by the *Hox* genes in the specification of neural lineages with axial-specific architectures and regional impact on behavior.

We wish to note that our analysis of *Hox* gene expression must not be seen as an argument that excludes other miRNA targets from playing central roles in the behaviors studied here. In the case of *miR-278*, *miR-8*, and *miR-980*, in addition to their effects on *Abd-B* expression, these miRNAs may impact behavior through effects on other target genes, acting on neural development and/or function. Furthermore, among the top 5% of genes enriched in targets for SR-miRNAs, we find genes with functional links to synaptic signaling (*e.g.*, *shaking B* and *Vesicular acetylcholine transporter*), neural development (*e.g.*, *elav* and *prospero*), and axon guidance (*e.g.*, *roundabout 3*), strongly suggesting that the expression of many other neural factors might be affected in SR-miRNA mutants (data not shown).

**Figure 5:**
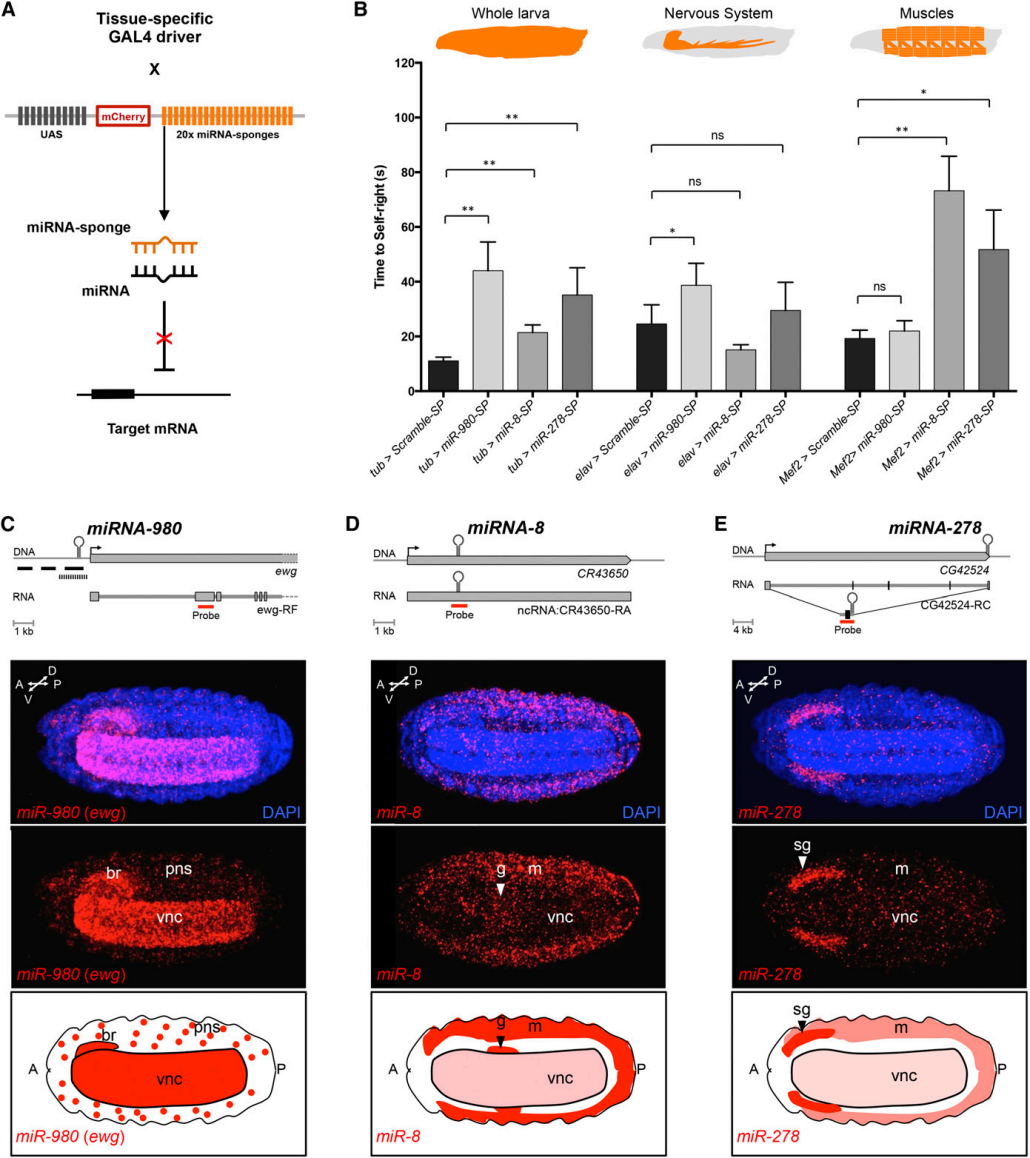
SR-miRNAs disrupt SR behavior through action in different tissues. (A) Schematic representation of miRNA-sponges (miR-SP) mode of action (based on Fulga *et al.* 2015): tissue-specific expression of mCherry constructs containing 20 miRNA-specific binding sites in the 3′-UTR. miRNAs will bind to the miR-SP, reducing the available RISC–miRNA complex that represses endogenous mRNA targets. (B) SR behavior in tissue-specific knockdown of miRNAs. miR-SPs were expressed in all larval tissues (*tubulin-Gal4*, left), nervous system (*elav^c155^-Gal4*, center), and muscle (*Mef2-Gal4*, right). Scramble-SP (UAS-sponge with a scrambled sequence; black bars) was used as control. *miR-980-SP* (light gray bars), *miR-8-SP* (gray bars), and *miR-278-SP* (dark gray bars) were used to knock down the miRNAs *miR-980*, *miR-8*, and *miR-278*, respectively. All three miR-SPs show a statistically significant delay in SR time compared with Scramble-SP when expressed ubiquitously (left, *tub* > *miR-SP*). Only *miR-980-SP* significantly recapitulated this SR delay when expressed exclusively in the nervous system (middle, *elav* > *miR-980-SP*), while *miR-8-SP* and *miR-278-SP* disrupted SR behavior when expressed in the muscle (right, *Mef2* > *miR-8-SP* and *Mef2* > *miR-278-SP*). Bars represent mean ± SEM; an average of 33 larvae per genotype were analyzed; Mann–Whitney *U*-test; ns, nonsignificant, * *P* < 0.05, ** *P* < 0.01). Additionally see Figure S6 in File S4 for parental line controls. (C–E) Embryonic expression of *miR-980*, *miR-8*, and *miR-278*. (C–E, top) Schematic representation of *miR-980*, *miR-8*, and *miR-278* loci. FISH RNA probes generated to detect the primary miRNA (pri-miRNA) transcripts are shown in red rectangles. Note that the full transcription unit of *miR-980* is unknown. None of the several probes designed and tested to detect the expression of the primary transcript of *miR-980* were successful (black rectangles represent probes used for conventional FISH and the black barred rectangle indicates the region of the 44 probes used for single-molecule FISH; data not shown). Given that the transcription start site of *erect wing* (*ewg*) is ∼500 bp downstream of *miR-980*, we used a probe targeting an exon present in all *ewg* mRNA isoforms (red rectangle) as a proxy for *miR-980* spatial expression. *miR-8* is located in the CR43650 long noncoding RNA, while *miR-278* is coded within the 3′-UTR of CG42524 mRNA isoform C. (C–E, middle) Spatial expression of *pri-miR-980* (*ewg*), *pri-miR-8*, and *pri-miR-278* obtained by RNA FISH using the red probes in the top panel. Whole-mounted late 16-stage embryos were imaged using confocal microscopy. (C) *pri-miR-980* (*ewg*) is expressed predominantly in the CNS and with punctual expression in PNS. (D) *pri-miR-8* is highly expressed in the muscle, in some neurons along the ventral nerve cord, and in the anterior gut. (E) *pri-miR-278* is strongly expressed in the salivary glands and in a few scattered muscle and CNS cells. DAPI is in blue. Anterior is to the left. br, brain; g, gut; m, muscle; pns, peripheral nervous system; sg, salivary glangs; vnc, ventral nerve cord. (C–E, bottom) Diagrams showing the expression patterns of *miR-980*, *miR-8*, and *miR-278* at late embryogenesis. Dark red represents high expression and light red depicts expression in small groups of cells.

To advance our understanding of the main tissues where miRNA functions are particularly critical, we launched two complementary series of experiments. First, we made use of miRNA-sponges, a molecular strategy that titers specific miRNAs away from natural mRNA targets by means of including a competing 3′-UTR sequence carrying multiple copies of miRNA target sequences (Figure 5A) (Fulga *et al.* 2015). We thus used specific miRNA-sponges to decrease the effects of miRNAs *miR-278*, *miR-8*, and *miR-980* in the whole larvae, the nervous system, or the muscle field (Figure 5B and Figure S6 in File S4). These experiments showed that an artificial decrease in the effects of these three miRNAs within the whole larvae leads to significant effects on SR time, confirming, with an independent approach, that normal function of these three miRNAs is required for SR behavior (Figure 5B, tub-Gal4 experiments). Interestingly, when the miRNA-sponges were selectively expressed in the CNS or muscle—two central tissues involved in movement control—results were distinct, depending on the miRNA under consideration: reduction of *miR-980* roles within the CNS led to a significant SR delay but no effects were observed when expressing a sponge for this miRNA in muscle (Figure 5B). In contrast, miRNA-sponges for *miR-8* and *miR-278* led to SR phenotypes only when expressed in the muscle but not the CNS (Figure 5B). These experiments suggest that different SR-miRNAs might exert their roles on SR through effects in distinct tissues.

Furthermore, RNA *in situ* hybridization experiments, aimed at detecting the broad transcriptional domains linked to the expression of these three miRNA genes, show that the three loci are active within the ventral nerve cord (vnc), yet showing different levels of expression (Figure 5, C–E). In addition, probes against *miR-8* and *miR-278* also showed detectable signal in the muscle (m) as well as in other tissues, including gut (g) and salivary glands (sg), for *miR-8* and *miR-278*, respectively (Figure 5, D and E). We must nonetheless note that these transcriptional domains provide only an approximation to the actual spatial distribution of mature miRNAs within the embryo. We also see that although mutation of miR-8 and miR-278 leads to a significant increase in Abd-B expression in the CNS, their expression levels in neural tissue are relatively low, opening the possibility that these miRNAs might exert their effects on Abd-B expression via intermediate factors.

All in all, information deduced from experiments with miRNA-sponges and miRNA expression analyses suggests that SR-miRNAs perform tissue specific roles: *miR-8* and *miR-278* may be acting primarily within muscle, while *miR-980* may be exerting its main effects in the nervous system. These data highlight the possibility that other SR-miRNAs might affect SR through roles in the nervous system as well as in other tissues.

Why might miRNAs have such pervasive roles in behavior? Given the relatively modest roles played by miRNAs in gene regulation (Baek *et al.* 2008; Selbach *et al.* 2008; Guo *et al.* 2010) the findings reported here are somewhat unexpected. Nonetheless, a closer look at the position of miRNAs within the gene regulatory networks controlling cellular features shows a different picture: miRNAs are network “hubs” with a high level of connectivity—formally, a high value of *out-degree k_o_* (Dorogovtsev and Mendes 2003)—achieved via their regulatory effects on hundreds of mRNA targets (Baek *et al.* 2008; Selbach *et al.* 2008). Although other factors within the cell—notably, transcription factors—also show high connectivity, their effects on target gene expression might be too pronounced for suitable behavioral analysis by means of null mutation, due to the resulting impact on cellular or organismal viability. In this context, we think that null mutations in miRNAs offer an unusual genetic setting that allows cells (and the organism) to retain viability, yet lead to significant effects on cellular dynamics via subtle but broad-based effects on the proteome. According to this view, miRNA effects may manifest in particularly pronounced form in the workings of the nervous system where cells must adhere to critical parameters to maintain suitable dynamics and functionality. Yet, effects need not be limited to neural tissue; for instance, miRNAs may affect neuromuscular communication or the biology of muscle cells themselves, the ultimate actuators of movement. We are currently exploring these possibilities mapping the neural circuits underlying SR and defining how miRNA expression relates to such circuitry with the view of further defining Benzer’s “focus” of action (Hotta and Benzer 1972) of miRNAs in regard to SR as well as regarding other behaviors in larvae and adults.

We have used an unbiased collection of miRNA mutants and a well-defined behavioral paradigm (SR) (Picao-Osorio *et al.* 2015) to test the possibility that miRNAs other than *miR-iab4* are involved in behavioral control either by inducing changes in developmental programs and/or physiological processes. Our experiments reveal that >40% of the miRNA mutant stocks in this collection have effects on SR behavior, unveiling a central regulatory role of miRNAs in the control of a complex behavior in *Drosophila*. To our best knowledge, this is the first time that miRNA regulation has been implicated at this scale in any behavior, in any animal system.

Our study also demonstrates that null miRNA mutations are powerful genetic tools to advance the understanding of the molecular mechanisms underlying complex behaviors in *Drosophila* and suggests that similar approaches to the one employed here could be applied to other species and behavioral paradigms with the prospect of advancing current models in behavioral genetics in other model organisms.

From a molecular perspective we anticipate that our work will contribute to the study of miRNA function *in vivo*, given that we reveal behavioral phenotypes for dozens of miRNA mutants in a genetically tractable system like *Drosophila*. These behavioral patterns can now be used as a phenotypic “read out” to evaluate the impact of molecular and cellular factors on the roles of miRNA regulation within the organism. In regards to behavior, our findings here, added to previous studies on the effects of single miRNA mutations on other behaviors including larval self-righting (Picao-Osorio *et al.* 2015), larval and adult feeding (Sokol and Ambros 2005; Vodala *et al.* 2012), adult climbing (Karres *et al.* 2007; Sokol *et al.* 2008; Liu *et al.* 2013; Chen *et al.* 2014; Verma *et al.* 2015), circadian rhythms (Luo and Sehgal 2012; Sun *et al.* 2015; Zhang *et al.* 2016), and adult startle locomotion (Yamamoto *et al.* 2008) demonstrate that the majority of *Drosophila* miRNA mutants (55%) tested to date lead to different types of behavioral defects. In addition to mutant analyses, a recent study using miRNA-sponges (an approach that allows reduction of miRNA activity) has shown that a decrease in miRNA function can affect memory formation in *Drosophila* adults (Busto *et al.* 2015) demonstrating that even a decrease in miRNA expression level might, in some instances, be sufficient to trigger effects on memory formation.

Based on the considerations mentioned above and the fact that the majority of the SR-miRNAs identified here are evolutionarily conserved between *Drosophila* and mammals (*i.e.*, 62.2%) (Ibáñez-Ventoso *et al.* 2008), we conclude that miRNAs are key molecular regulators of the genetic programs underlying behavior in *Drosophila* and are most likely to play similar roles in other animal species including humans.

## Supplementary Material

Supplemental material is available online at www.genetics.org/lookup/suppl/doi:10.1534/genetics.116.195776/-/DC1.
