# Supplementary material for: Pervasive Behavioral Effects of MicroRNA Regulation in Drosophila
Source: Genetics. 2017 May 2;206(3):1535–48. doi: 10.1534/genetics.116.195776 (PMC5500149; doi:10.1534/genetics.116.195776)
Supplement: Supplementary file 1 [file 1535FileS1.docx]

| **miRNA stock** | **BDSC number/Origin** | **Detailed genotype** |
| --- | --- | --- |
| **w^1118^** | 5905 | w[1118] |
| **yw** | 1495 | y[1] w[1] |
| **Bantam** | Stephen Cohen’s Lab  (Brennecke *et al.,* 2003) | w[1118];bantam^Δ1^/TM3, P{w[+mC]=GAL4-twi.G}2.3, P{UAS-2xEGFP}AH2.3, Sb[1] Ser[1] |
| **mir-1** | 58879 | w[*]; mir-1[KO]/CyO, P{w[+mC]=GAL4-twi.G}2.2, P{UAS-2xEGFP}AH2.2 |
| **mir-10** | 58880 | w[*]; TI{w[+mW.hs]=GAL4}mir-10[KO] |
| **mir-100/let-7/125** | 58881 | w[*]; Df(2L)let-7-C[KO1], TI{w[+m*]=TI}CG10283[K01]/CyO, P{w[+mC]=GAL4-Kr.C}DC3, P{w[+mC]=UAS-GFP.S65T}DC7 |
| **mir-1000** | 58882 | w[*]; TI{TI}mir-1000[KO]/TM3, P{w[+mC]=GAL4-twi.G}2.3, P{UAS-2xEGFP}AH2.3, Sb[1] Ser[1] |
| **mir-1003** | 58883 | w[*]; TI{TI}mir-1003[KO]/TM3, P{w[+mC]=GAL4-twi.G}2.3, P{UAS-2xEGFP}AH2.3, Sb[1] Ser[1] |
| **mir-1010** | 58886 | w[*]; TI{TI}mir-1010[KO]/TM3, P{w[+mC]=GAL4-twi.G}2.3, P{UAS-2xEGFP}AH2.3, Sb[1] Ser[1] |
| **mir-1017** | 58889 | w[*]; TI{TI}mir-1017[KO]/TM3, P{w[+mC]=GAL4-twi.G}2.3, P{UAS-2xEGFP}AH2.3, Sb[1] Ser[1] |
| **mir-11** | 58890 | w[*]; TI{TI}-mir11[KO.w-] |
| **mir-124** | Sun *et al.,* 2003; Zhang *et al*., 2007 (1) | w[*]; mir-124[KO]/CyO |
| **mir-133** | 58892 | w[*]; TI{w[+mW.hs]=TI}mir-133[KO]/CyO, P{w[+mC]=GAL4-twi.G}2.2, P{UAS-2xEGFP}AH2.2 |
| **mir-137** | 58893 | w[*]; TI{w[+mW.hs]=TI}mir-137[KO] |
| **mir-13b-2** | 58894 | y[1] w[*] TI{TI}mir-13b-2[KO] |
| **mir-14** | 58895 | w[*]; mir-14[Delta1]/CyO, P{w[+mC]=GAL4-Kr.C}DC3, P{w[+mC]=UAS-GFP.S65T}DC7 |
| **mir-184** | 58896 | w[*]; TI{w[+mW.hs]=TI}mir-184[KO]/CyO, P{w[+mC]=GAL4-twi.G}2.2, P{UAS-2xEGFP}AH2.2 |
| **mir-190** | 58897 | w[*]; TI{TI}mir-190[KO]/TM3, P{w[+mC]=GAL4-twi.G}2.3, P{UAS-2xEGFP}AH2.3, Sb[1] Ser[1] |
| **mir-193** | 58898 | w[*]; TI{w[+mW.hs]=GAL4}mir-193[KO]/TM3, P{w[+mC]=GAL4-twi.G}2.3, P{UAS-2xEGFP}AH2.3, Sb[1] Ser[1] |
| **mir-210** | 58899 | y[1] w[*] TI{w[+mW.hs]=GAL4}mir-210[KO] |
| **mir-219** | 58900 | w[*]; TI{w[+mW.hs]=TI}mir-219[KO]/TM3, P{w[+mC]=GAL4-twi.G}2.3, P{UAS-2xEGFP}AH2.3, Sb[1] Ser[1] |
| **mir-252** | 58901 | w[*]; TI{TI}mir-252[KO]/TM3, P{w[+mC]=GAL4-twi.G}2.3, P{UAS-2xEGFP}AH2.3, Sb[1] Ser[1] |
| **mir-263a** | 58902 | w[*]; TI{w[+mW.hs]=TI}bft[Delta263a]/CyO, P{w[+mC]=GAL4-twi.G}2.2, P{UAS-2xEGFP}AH2.2 |
| **mir-263b** | 58903 | w[*]; TI{TI}mir-263b[Delta] |
| **mir-274** | 58904 | w[*]; TI{TI}mir-274[KO]/TM3, P{w[+mC]=GAL4-twi.G}2.3, P{UAS-2xEGFP}AH2.3, Sb[1] Ser[1] |
| **mir-275/305** | 58905 | w[*]; Df(2L)mir-275-305-KO, TI{w[+mW.hs]=TI}mir-275-305-KO/CyO, P{w[+mC]=GAL4-twi.G}2.2, P{UAS-2xEGFP}AH2.2 |
| **mir-276a** | 58906 | w[*]; TI{w[+mW.hs]=TI}mir-276a[KO]/TM3, P{w[+mC]=GAL4-twi.G}2.3, P{UAS-2xEGFP}AH2.3, Sb[1] Ser[1] |
| **mir-276b** | 58907 | w[*]; TI{TI}mir-276b[KO]/TM3, P{w[+mC]=GAL4-twi.G}2.3, P{UAS-2xEGFP}AH2.3, Sb[1] Ser[1] |
| **mir-277/34** | 58908 | w[*]; Df(3R)mir-277-34-KO, TI{w[+mW.hs]=TI}mir-277-34-KO/TM3, P{w[+mC]=GAL4-twi.G}2.3, P{UAS-2xEGFP}AH2.3, Sb[1] Ser[1] |
| **mir-278** | 58909 | w[*]; TI{w[+mW.hs]=TI}mir-278[KO] |
| **mir-281-1/281-2** | 58910 | w[*]; Df(2R)mir-281-1-281-2-KO/CyO, P{w[+mC]=GAL4-twi.G}2.2, P{UAS-2xEGFP}AH2.2 |
| **mir-282** | 58911 | w[*]; TI{w[+mW.hs]=TI}mir-282[KO]/TM3, P{w[+mC]=GAL4-twi.G}2.3, P{UAS-2xEGFP}AH2.3, Sb[1] Ser[1] |
| **mir-283** | 58912 | w[*] TI{TI}mir-283[KO] |
| **mir-284** | 58913 | w[*]; TI{w[+mW.hs]=TI}mir-284[KO]/TM3, P{w[+mC]=GAL4-twi.G}2.3, P{UAS-2xEGFP}AH2.3, Sb[1] Ser[1] |
| **mir-285** | 58914 | w[*]; TI{w[+mW.hs]=TI}mir-285[KO] |
| **mir-2a-2/2a-1/2b-2** | 59032 | w[*]; Df(2L)mir-2a-2-2a-1-2b-2-KO/CyO, P{w[+mC]=GAL4-twi.G}2.2, P{UAS-2xEGFP}AH2.2 |
| **mir-2b-1** | 58915 | w[*]; TI{w[+mW.hs]=TI}mir-2b-1[KO] |
| **mir-2c/13a/13b-1** | 58916 | w[*]; Df(3R)mir-2c-13a-13b-1-KO, TI{w[+mW.hs]=TI}mir-2c-13a-13b-1-KO |
| **mir-304** | 58918 | w[*] TI{TI}mir-304[KO] |
| **mir-306/79/9b** | 58919 | w[*]; Df(2L)mir-306-79-9b-KO/CyO, P{w[+mC]=GAL4-twi.G}2.2, P{UAS-2xEGFP}AH2.2 |
| **mir-307a/307b** | 58920 (1) | w[*]; Df(2R)mir-307a-307b-KO/CyO |
| **mir-308** | 58921 | w[*]; TI{TI}mir-308[KO]/CyO, P{w[+mC]=GAL4-twi.G}2.2, P{UAS-2xEGFP}AH2.2 |
| **mir-310/311/312/313** | 58923 | w[*]; Df(2R)mir-310-311-312-313 P{ry[+t7.2]=neoFRT}42D/CyO, P{w[+mC]=GAL4-twi.G}2.2, P{UAS-2xEGFP}AH2.2 |
| **mir-314** | 58924 | w[*]; TI{w[+mW.hs]=TI}mir-314[KO] |
| **mir-316** | 58925 | w[*]; TI{w[+mW.hs]=TI}mir-316[KO]/TM3, P{w[+mC]=GAL4-twi.G}2.3, P{UAS-2xEGFP}AH2.3, Sb[1] Ser[1] |
| **mir-317** | 58926 (1) | w[*]; TI{w[+mW.hs]=TI}mir-317[KO]/TM3, Sb[1] |
| **mir-318** | 58927 | w[*]; TI{w[+mW.hs]=TI}mir-318[KO]/TM3, P{w[+mC]=GAL4-twi.G}2.3, P{UAS-2xEGFP}AH2.3, Sb[1] Ser[1] |
| **mir-31a** | 58928 | w[*]; TI{w[+mW.hs]=TI}mir-31a[KO] |
| **mir-31b** | 58929 | w[*] TI{TI}mir-31b[KO] |
| **mir-33** | 58930 | w[*]; TI{TI}mir-33[KO] |
| **mir-375** | 58931 | w[*]; TI{w[+mW.hs]=GAL4}mir-375[KO]/CyO, P{w[+mC]=GAL4-twi.G}2.2, P{UAS-2xEGFP}AH2.2 |
| **mir-8** | 58932 | w[*]; mir-8[Delta2]/CyO, P{w[+mC]=GAL4-Kr.C}DC3, P{w[+mC]=UAS-GFP.S65T}DC7 |
| **mir-87** | 58934 | w[*]; TI{w[+mW.hs]=TI}mir-87[KO]/CyO, P{w[+mC]=GAL4-twi.G}2.2, P{UAS-2xEGFP}AH2.2 |
| **mir-927** | 58935 | y[1] w[*] TI{TI}mir-927[KO] |
| **mir-929** | 58936 | w[*]; TI{TI}mir-929[KO]/TM3, P{w[+mC]=GAL4-twi.G}2.3, P{UAS-2xEGFP}AH2.3, Sb[1] Ser[1] |
| **mir-92a** | 58937 | w[*]; TI{TI}mir-92a[KO] |
| **mir-92b** | 58938 (1) | w[*]; TI{w[+mW.hs]=TI}mir-92b[KO]/TM3, Sb[1] |
| **mir-932** | 58939 | w[*]; TI{TI}mir-932[KO]/CyO, P{w[+mC]=GAL4-twi.G}2.2, P{UAS-2xEGFP}AH2.2 |
| **mir-955** | 58940 | w[*]; TI{w[+mW.hs]=GAL4}mir-955[KO] |
| **mir-956** | 58941 | w[*]; TI{w[+mW.hs]=GAL4}mir-956[KO]/TM3, P{w[+mC]=GAL4-twi.G}2.3, P{UAS-2xEGFP}AH2.3, Sb[1] Ser[1] |
| **mir-957** | 58942 | w[*]; TI{w[+mW.hs]=TI}mir-957[KO] |
| **mir-958** | 58943 | w[*]; TI{w[+mW.hs]=TI}mir-958[KO] |
| **mir-959/960/961/962** | 58944 | w[*]; Df(2L)mir-959-960-961-962-KO |
| **mir-963/964** | 58945 | w[*]; Df(2L)mir-963-964-KO/CyO, P{w[+mC]=GAL4-twi.G}2.2, P{UAS-2xEGFP}AH2.2 |
| **mir-965** | 58946 (1) | w[*]; TI{TI}mir-965[KO1]/CyO |
| **mir-966** | 58947 | w[*]; TI{TI}mir-966[KO]/CyO, P{w[+mC]=GAL4-twi.G}2.2, P{UAS-2xEGFP}AH2.2 |
| **mir-968/1002** | 58949 | w[*]; Df(2L)mir-968-1002-KO, TI{w[+mW.hs]=GAL4}mir-968-1002-KO/CyO, P{w[+mC]=GAL4-twi.G}2.2, P{UAS-2xEGFP}AH2.2 |
| **mir-970** | 58951 | w[*] TI{TI}mir-970[KO] |
| **mir-971** | 58952 | w[*] TI{w[+mW.hs]=GAL4}mir-971[KO] |
| **mir-972/973/974** | 58953 | Df(1)mir-972-973-974-KO, y[1] w[*] TI{w[+mW.hs]=GAL4}mir-972-973-974-KO |
| **mir-975/976/977** | 58954 | Df(1)mir-975-976-977-KO, w[*] TI{w[+mW.hs]=GAL4}mir-975-976-977-KO |
| **mir-980** | 58955 | TI{TI}mir-980[KO] w[*] |
| **mir-981** | 58956 (1) | TI{TI}mir-981[KO] w[*]/FM6 |
| **mir-982/303** | 58957 | Df(1)mir-982-303-KO, w[*] TI{w[+mW.hs]=TI}mir-982-303-KO |
| **mir-984/983-1/983-2** | 58958 | Df(1)mir-984-983-1-983-2-KO, w[*] |
| **mir-986** | 58959 | w[*]; TI{TI}mir-986[KO]/CyO, P{w[+mC]=GAL4-twi.G}2.2, P{UAS-2xEGFP}AH2.2 |
| **mir-987** | 58960 | w[*]; TI{w[+mW.hs]=GAL4}mir-987[KO]/CyO, P{w[+mC]=GAL4-twi.G}2.2, P{UAS-2xEGFP}AH2.2 |
| **mir-988** | 58961 | w[*]; TI{TI}mir-988[KO] |
| **mir-989** | 58962 | w[*]; TI{w[+mW.hs]=TI}mir-989[KO]/CyO, P{w[+mC]=GAL4-twi.G}2.2, P{UAS-2xEGFP}AH2.2 |
| **mir-990** | 58963 | w[*]; TI{TI}mir-990[KO]/CyO, P{w[+mC]=GAL4-twi.G}2.2, P{UAS-2xEGFP}AH2.2 |
| **mir-994** | 58964 | w[*]; TI{w[+mW.hs]=TI}mir-994[KO]/TM3, P{w[+mC]=GAL4-twi.G}2.3, P{UAS-2xEGFP}AH2.3, Sb[1] Ser[1] |
| **mir-995** | 58965 | w[*]; TI{TI}mir-995[KO]/TM3, P{w[+mC]=GAL4-twi.G}2.3, P{UAS-2xEGFP}AH2.3, Sb[1] Ser[1] |
| **mir-999** | 58966 | w[*]; TI{TI}mir-999[KO] |
| **mir-9c** | 58967 | w[*]; TI{TI}mir-9c[KO]/CyO, P{w[+mC]=GAL4-twi.G}2.2, P{UAS-2xEGFP}AH2.2 |
| **mir-iab-4/iab-8** | Bender, 2008 | w[1118]; ΔmiR-iab-4/iab-8/TM3, P{w[+mC]=GAL4-twi.G}2.3, P{UAS-2xEGFP}AH2.3, Sb[1] Ser[1] |

(1) Stocks re-balanced with fluorescent chromosomal balancers:

TM3, P{w[+mc]=GAL4-twi.G}2.3, P{UAS-2xEGFP}AH2.3, Sb[1] (BDSC #6663)

CyO, P{w[+mc]=GAL4-twi.G}2.2, P{UAS-2xEGFP}AH2.2 (BDSC #6662)

FM7c, P{w[+mc]=GAL4-twi.G}108.4, P{UAS-2xEGFP}AX (BDSC #6873)
