# Supplementary material for: Pervasive Behavioral Effects of MicroRNA Regulation in Drosophila
Source: Genetics. 2017 May 2;206(3):1535–48. doi: 10.1534/genetics.116.195776 (PMC5500149; doi:10.1534/genetics.116.195776)
Supplement: Supplementary file 4 [file 1535FileS4.pdf]

Pervasive behavioural effects of microRNA regulation in *Drosophila*

Joao Picao-Osorio[#], Ines Lago-Baldaia[#], Pedro Patraquim and Claudio R. Alonso*

Sussex Neuroscience,
School of Life Sciences,
University of Sussex,
Brighton BN1 9QG
United Kingdom

[#]*Equal contribution*

**Correspondence to:*

Claudio R. Alonso
c.alonso@sussex.ac.uk
+44 1273 876621
+44 794 493 0572

# - Supplemental Figures -

Figure S1

Picao-Osorio *et al.*

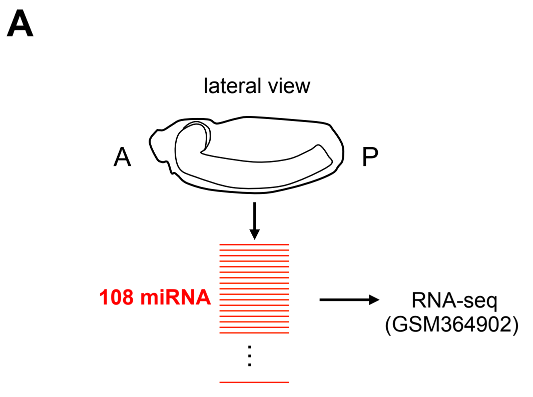

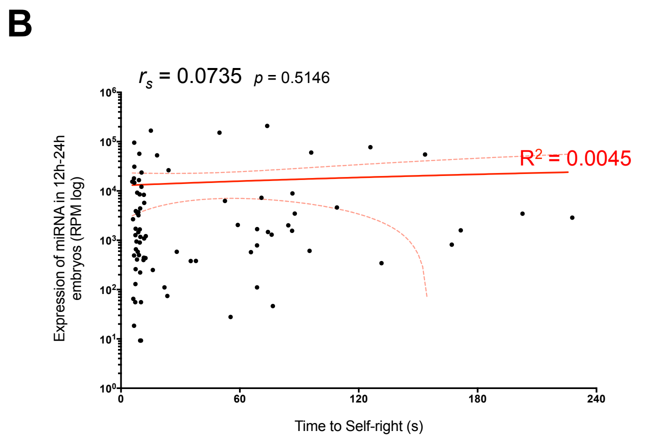

Figure Supplementary 1. Association between miRNA expression level and time to SR .(**A**) Schematic representation of the 108 miRNA expression levels from 12h-24h of embryogenesis (see S2 File; (Chung *et al.* 2008)). (B) Plot showing the association between the expression level of the 108 miRNAs studied at 12h-24h of embryogenesis (y-axis) and time to self-right (x-axis). Linear regression (R^2=0.0045) and 95% confidence interval in red line and doted line, respectively. The Spearman coefficient (r_s) and p value are shown. There is no significant correlation between miRNA expression and the SR delay (r_s=0.0735; p=0.5146).

Figure S2
Picao-Osorio *et al.*

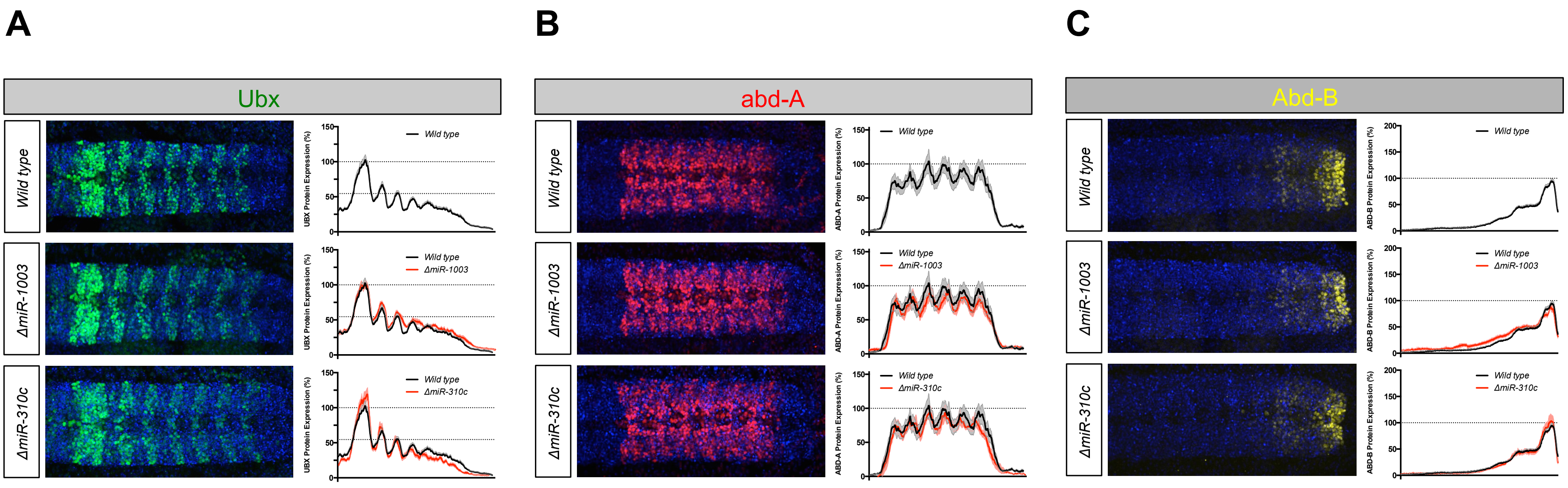

Figure Supplementary 2. Protein expression of Hox genes Ubx, Abd-A and Abd-B in *miR-1003* and *miR-310c* mutant embryos (A-C, left) Protein expression of Ubx (A, green), Abd-A (B, red) and Abd-B (C, yellow) in ventral nerve cords of *wild-type* and mutants for *miR-1003* and *miR-310c* embryos at late 16 stage. (A-C, right) Profile quantification along the A-P axis for the three Hox proteins in the *wild-type* (mean in black line and SEM in grey) and in the *miRNA* mutants (mean in red line and SEM in lighter red). These *miRNA* mutants did show any significant protein expression level difference for any of the three Hox proteins. N=10 embryos per genotype for each immunostaining. DAPI in blue. Anterior is to the left.

Figure S3
Picao-Osorio *et al.*

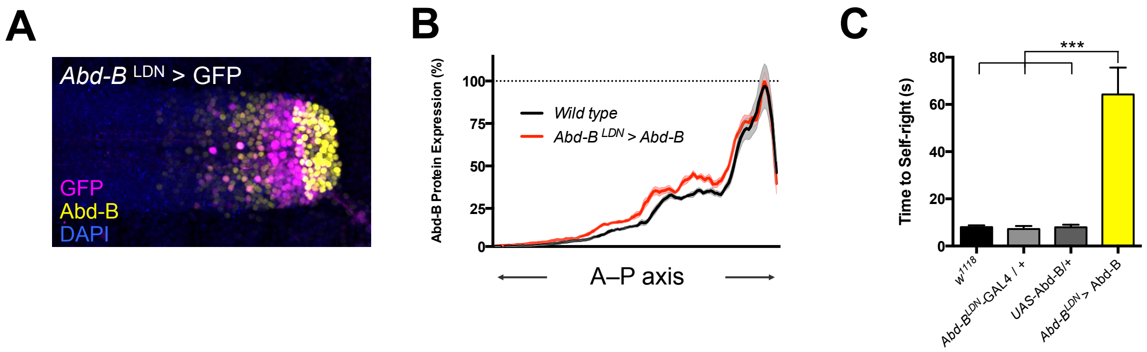

Figure Supplementary 3. Overexpression of Abd-B disrupts SR behaviour (**A**) Expression pattern of *Abd-B*^{LDN}-GAL4 driver (GFP, magenta), in respect to the endogenous pattern of Abd-B protein expression (yellow) in dissected embryonic ventral nerve cord. DAPI in blue and anterior is to the left. (**B**) Quantification of Abd-B expression profile along the A-P axis in dissected embryonic nerve cords of *wild-type* (w^{1118}, mean in black and SEM in grey) and Abd-B overexpression (*Abd*-B^{LDN}> Abd-B mean in magenta and SEM in light magenta) (N = 9 embryos per genotype). (**C**) Significant delay in time to SR in larvae overexpressing Abd-B (*Abd-B*^{LDN} > Abd-B, yellow bars) in comparison with *wild-type* (w^{1118}) and parental lines (*Abd-B*^{LDN}-GAL4/+, light grey bar, and *UAS*-Abd-B/+ in dark grey) (mean ± SEM; an average of 20 larvae per genotype were analysed; Mann-Whitney *U* test with Bonferroni correction, *** $p <$ 0.001).

Figure S4
Picao-Osorio *et al.*

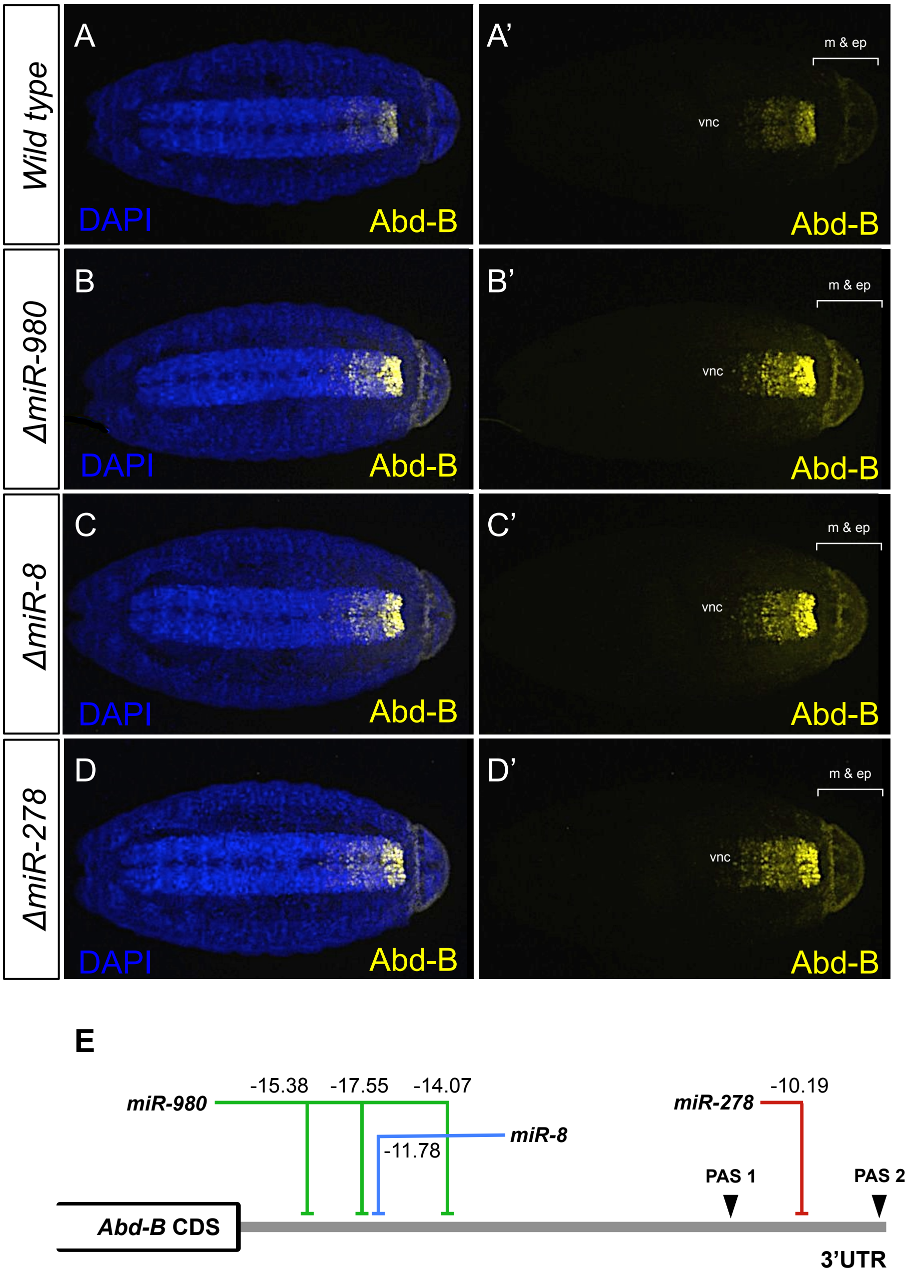

Figure Supplementary 4. Whole embryo Abd-B protein expression in *miR-980*, *miR-8*, *miR-iab4/iab8* and *miR-278* mutants. (**A-D**) Abd-B protein expression (yellow) in whole embryos of *wild-type* and mutants for *miR-980*, *miR-8* and *miR-278* at late 16 stage counterstained with DAPI in blue (A-D). The increase of Abd-B in the ventral nerve cord (vnc) of these mutants is accompanied by increase in muscle and epiderm (m & ep) of the most posterior segments (indicated with brackets). (**E**) Schematic representation of *miR-980* (green), *miR-8* (blue) and *miR-278* (red) predicted binding sites to the longest *Abd-B* 3'UTR annotated (BDGP6, see Materials and Methods), according to the PITA algorithm to predict miRNA target sites (Kertesz *et al*.2007).. Each individual site ΔΔG score from PITA is indicated. Note that the more negative a ΔΔG score is, the more probable is the targeting of a miRNA to a site in the 3'UTR. The two alternative polyadenylation signals (PAS) are represented with black arrowheads.

Figure S5

Picao-Osorio *et al.*

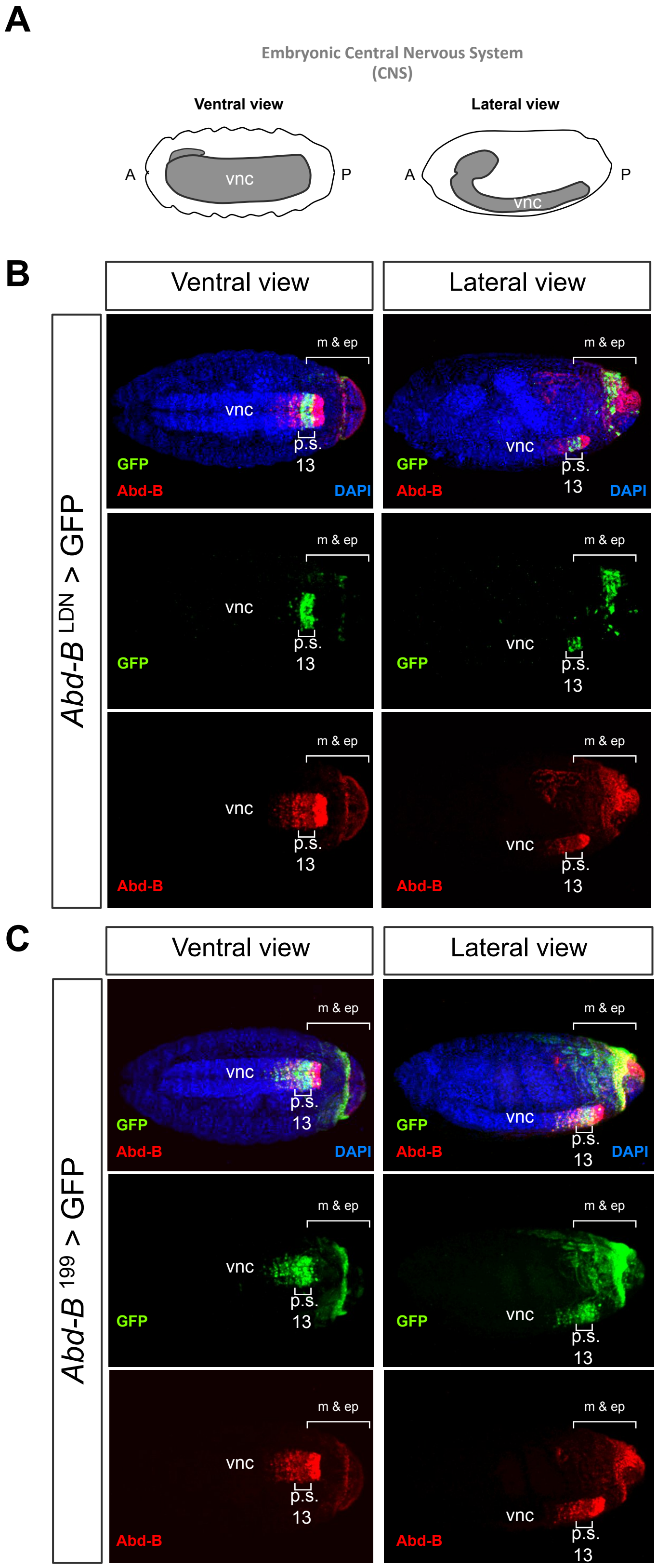

Figure Supplementary 5. Whole embryo expression pattern of *Abd-B*-Gal4 drivers. (**A**) Schematic representation of the ventral and lateral views of late 16 stage embryos. The embryonic central nervous system is represented in grey. (**B-C**) Expression pattern of *Abd-B^{LDN}*-GAL4 (B) and *Abd-B^{199}*-GAL4 (C) drivers (GFP, green) of whole mounted embryos in respect to the endogenous pattern of Abd-B protein expression (red) counterstained with DAPI (blue). (B) *Abd-B^{LDN}*-GAL4 drives expression in parasegment (p.s.) 13 of the embryonic ventral nerve cord (vnc), muscle (m) and epidermis (ep). (C) *Abd-B^{199}*-GAL4 drives high expression in p.s. 13 of the embryonic vnc, m and ep, and lower expression in p.s 12 of the same tissues. Anterior is to the left.

Figure S6

Picao-Osorio *et al.*

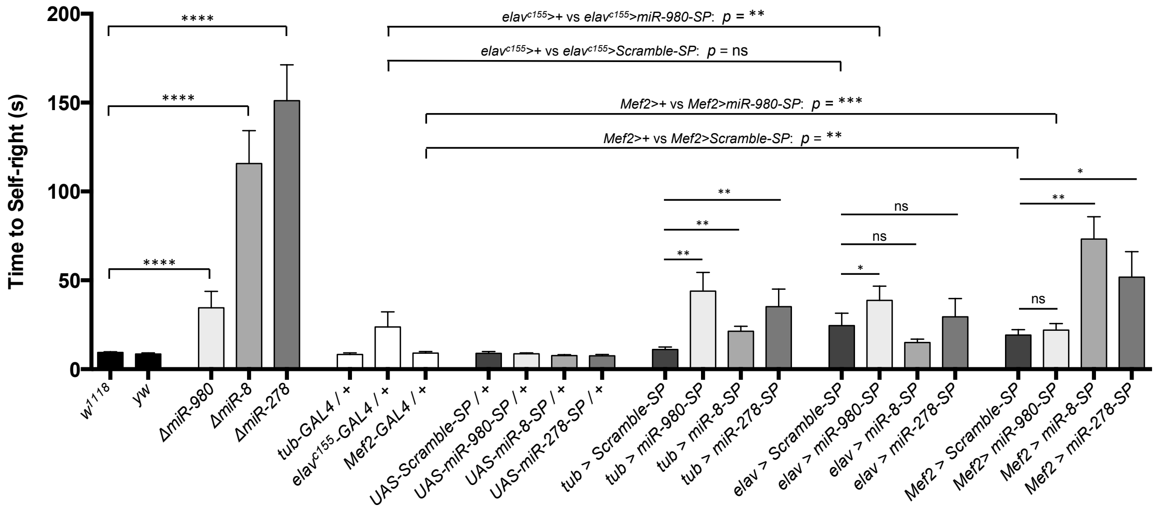

Figure Supplementary 6. SR time of controls for miR-sponges experiment (Fig. 5B). (A) Time to self-right in seconds (s) of the two genetic background lines (w^{1118} and *yw*, black bars), as well as all the parental lines (GAL4/+ and *UAS-miR-SP*/+), used to obtained the experimental larvae (GAL4>*UAS-miR-SP*, shown in Fig. 5B). Since these crosses and the developing embryos were maintained at 29°C for maximal Gal4 activity, the miRNA KO mutants were re-analysed at this temperature. At 29°C the three SR times were slightly decreased compared when embryonic development was conducted at 25°C (Fig. 1B) but still within the same magnitude of effect. Gal4 drivers (GAL4/+) are represented with white bars. All genotypes related with *miR-980* are represented in light grey, *miR-8* in medium grey, and *miR-278* in dark grey. *Scramble-SP* related genotypes are the darkest grey bars. Bars represent mean ± SEM; an average of 35 larvae per genotype were analysed; Mann-Whitney *U* test; (ns) non-significant $p > 0.05$, * $p < 0.05$, ** $p < 0.01$, *** $p < 0.001$, **** $p < 0.0001$).
