# Supplementary material for: Pervasive Behavioral Effects of MicroRNA Regulation in Drosophila
Source: Genetics. 2017 May 2;206(3):1535–48. doi: 10.1534/genetics.116.195776 (PMC5500149; doi:10.1534/genetics.116.195776)
Supplement: Supplementary file 5 [file 1535FileS5.docx]

**Pervasive behavioural effects of microRNA regulation in *Drosophila***

Joao Picao-Osorio^#^, Ines Lago-Baldaia^#^, Pedro Patraquim and Claudio R. Alonso*

*Sussex Neuroscience,*

*School of Life Sciences,*

*University of Sussex,*

*Brighton BN1 9QG*

*United Kingdom*

^#^*Equal contribution*

**Correspondence to:*

Claudio R. Alonso

[*c.alonso@sussex.ac.uk*](mailto:c.alonso@sussex.ac.uk)

+44 1273 876621

+44 794 493 0572

**- Legends of Supplementary Files –**

**Supplementary File 01.** List of miRNA mutants and control fly stocks with source of origin and detailed genotype.

**Supplementary File 02.** List of read-per-million counts from miRNA RNA-seq data of *Drosophila melanogaster* 12-24hr embryos (Canton S strain) (NCBI GEO accession GSM364902). The coverage of miRNAs analysed in the behaviour screen was calculated by dividing the total number of reads of miRNAs analysed by the total number of all miRNA reads present in the library.

**Supplementary File 03.** SR-miRNA target prediction for the BX-C genes. The longest annotated 3’UTR of the three BX-C genes (Ubx, abd-A and Abd-B) were scanned for predicted miRNA target sites of the 33 SR-miRNA. The results from two independent platforms, PITA (Kertesz et al. 2007) and miRanda (Betel et al. 2008), were overlapped. miRNAs present in both lists were allocated the value 1. SR-miRNAs targeting at least one gene are highlighted in light grey.
